# [The innovation characteristics of person-centred care as perceived by healthcare professionals: an interview study employing a deductive-inductive content analysis guided by the consolidated framework for implementation research

**DOI:** 10.1186/s12913-021-06942-y

**Published:** 2021-09-03

**Authors:** Helena Fridberg, Lars Wallin, Malin Tistad

**Affiliations:** 1 School of Health and Welfare, Dalarna University, Falun, Sweden; 2 Institute of Health and Care Sciences and University of Gothenburg Centre for Person-Centred Care, Sahlgrenska Academy at the University of Gothenburg, Gothenburg, Sweden; 3 Department of Neurobiology, Care Sciences and Society, Karolinska Institute, Stockholm, Sweden

**Keywords:** Consolidated framework for implementation research, Deductive, Implementation science, Inductive, Innovation, Person-centred care, Qualitative content analysis

## Abstract

**Background:**

Person-centred care (PCC) is promoted as an innovation that will improve patients’ rights and increase their participation in healthcare. Experience shows that the implementation of PCC is challenging and often results in varying levels of adoption. How health care professionals (HCPs) perceive an innovation such as PCC is an important factor to consider in implementation. Yet, such studies are scarce. Thus, in a sample of healthcare units in a region in Sweden, involved in a transition to PCC, we aimed to investigate HCPs’ perceptions of PCC.

**Methods:**

An interview study was conducted in 2018 during the implementation of PCC with HCPs (*n* = 97) representing diverse vocational roles in six healthcare contexts. Data were collected via focus groups (*n* = 15), dyadic interviews (*n* = 5), and individual interviews (*n* = 22) and analysed using a deductive–inductive content analysis. The deductive approach was guided by the Consolidated Framework for Implementation Research (CFIR), followed by an inductive analysis to describe HCPs’ in-depth perceptions of PCC in relation to each of the CFIR constructs.

**Results:**

Eight constructs from two of the CFIR domains, Intervention characteristics and Inner setting, were used to code HCPs’ perceptions of PCC. One construct, Observability, was added to the coding sheet to fully describe all the data. The constructs Relative advantage, Complexity, Compatibility, Observability, and Available resources were discussed in depth by HCPs and resulted in rich and detailed data in the inductive data analysis. This analysis showed large variations in perceptions of PCC among HCPs, based on factors such as the PCCs ethical underpinnings, its operationalisation into concrete working routines, and each HCPs’ unique recognition of PCC and the value they placed on it.

**Conclusions:**

We identified nine CFIR constructs that seem pertinent to HCPs’ perceptions of PCC. HCPs report an array of mixed perceptions of PCC, underlining its complex nature. The perceptions are shaped by a range of factors, such as their individual understandings of the concept and the operationalisation of PCC in their local context. Stakeholders in charge of implementing PCC might use the results as a guide, delineating factors that may be important to consider in a wide range of healthcare contexts.

**Supplementary Information:**

The online version contains supplementary material available at 10.1186/s12913-021-06942-y.

## Background

New evidence-based innovations are challenging to implement and embed in clinical practice and there is an urgent need for more studies examining the factors that impact upon these processes in order to ensure their success [1]. How adopters perceive an innovation is a factor that is known to strongly affect implementation outcomes [1, 2].

A paradigm shift is apparent in healthcare settings across the globe, with patients being given more power and trusted as active agents participating in, and making decisions about, their healthcare together with HCPs [3, 4]. PCC is a key concept in this paradigm shift and is promoted by policymakers and stakeholders in both healthcare and the wider society as a means of delivering high quality care that is ethical, equitable and promotes healthcare on patients’ own terms, with the ultimate goal of striving for a meaningful life [3, 5, 6]. PCC as a concept is underpinned by ethical and philosophical values whereby each patient should be regarded as a person with individual needs, wishes, and resources based on their lived experiences [7, 8]. There is no agreed-upon definition of PCC, which complicates the concept’s operationalisation into practice and makes it difficult to aggregate findings from different studies [9, 10]. Thus, PCC suffers from a plethora of definitions as well as a multitude of operationalisations, resulting in an array of activities at the individual level, between HCPs and patients, within any given organisation, and across organisations [5, 10]. Researchers at The Gothenburg University Centre for Person-centred Care (GPCC) have developed an ethical approach aiming to help HCPs to work more in line with PCC via three routines that are thought to strengthen the partnership between HCPs and patients [8]:

- *Initiating the partnership* through listening to patients’ narratives in order to understand their wishes, capabilities, and resources for illness management and what really matters in their everyday lives.
- *Working the partnership* through the co-creation of care between the HCP and the patient. Medical diagnoses and treatments are discussed in conjunction with the patient’s wishes and priorities in order to gain a clear understanding of the patient’s situation, leading to a health plan with goals based on the patient’s priorities and collaboration with HCPs.
- *Safeguarding the partnership* entails documentation of the health plan in order to support continuity of care. The health plan is revised when changes are being made and is accessible to patients and all HCPs who are part of their healthcare [8, 11].

The key characteristics of the implementation object, the innovation (sometimes denoted intervention), is part of most implementation frameworks, models, and theories that aim to guide, facilitate, and explain implementation efforts [12]. An innovation is characterised (for the purpose of this study) as the new practice being implemented. How HCPs perceive an innovation in relation to their working context has been shown to be a crucial determinant of the outcome of the implementation [13]. An innovation should not be regarded as inheriting fixed attributes that are perceived similarly by all the intended adopters [13]. It is imperative to understand that HCPs use their unique lived experience, knowledge, values, and so on when they approach and work with or around an innovation [13, 14]. Moreover, and complicating things further, this individual process is also affected by others, such as managers’ and HCPs’ peers take-up of the innovation in the workplace [5, 13, 15].

### Theoretical framework

The Consolidated Framework for Implementation Research (CFIR) is a determinant framework based on a synthesis of previous frameworks and research that has been refined to deliver operationally defined constructs [16]. It is organised into five domains with a total of 39 constructs aiming to describe factors that will influence implementation efforts. These domains are: *Intervention characteristics, Outer setting, Inner setting, Characteristics of the individuals,* and *Process.* The founders of the CFIR did not specify a hierarchy or interactions between the constructs and they recommend that researchers choose constructs deliberately, based on their relevance, and adapt them to fit each specific innovation and its context [16, 17]. We chose to use the term innovation to denote the practice to be implemented (in this case PCC), as intervention could too easily be mistaken for the implementation activity itself. Consequently, the domain intervention characteristics is denoted innovation characteristics in this study.

There is a lack of knowledge concerning efforts to implement innovations in everyday care without support from researchers. By gaining a deeper insight into the factors that are important for broad implementation across healthcare contexts, successful and promising strategies can be identified and shared within and across regions in order to improve the implementation of PCC. How target groups perceive the innovation should be of interest to all stakeholders wishing to increase the impact of implementation strategies. Thus, the aim of this cross-sectional study is to describe how HCPs perceive PCC in relation to their own context through the lens of the CFIR.

## Methods

This observational study is conducted within the frame of a larger project seeking to increase knowledge about the implementation of PCC carried out as part of everyday care without any input from researchers, i.e., a natural experiment.

### Setting

#### Contextual background

The study took place in a healthcare region in central Sweden. The region has six hospitals and approximately 30 primary healthcare units providing healthcare to around 280,000 inhabitants across an area of 28,000 km^2^. In 2015, policymakers in the region made a political decision to elicit more PCC. Staff at the Department for Development developed a strategy at the regional level to support the transition to more PCC. They chose to promote PCC in line with GPCC’s approach. The implementation processes in care settings, supported by the regional support strategy, are examples of implementation as usual, here defined as current implementation efforts in care settings with no involvement of researchers.

#### Support strategy and presentation of the innovation at the regional level

The support strategy included an offer to all healthcare units in the region to participate in a series of three, full-day learning seminars. The initial top-down approach aimed to disseminate knowledge and provide initial support to healthcare units in their implementation work. The learning seminars were free of charge and included lunch for all participants. The senior and frontline managers at each healthcare unit decided which and how many HCPs should take part in the seminars. They were strongly encouraged to enrol a wide selection of HCPs to enhance team discussions. The learning seminars included lectures, workshops, and discussions. Lectures covered a broad spectrum of topics ranging from the ethical underpinnings of PCC, results from trials on PCC as well as the three core routines brought forward by GPCC; initiating, working and safeguarding the partnership. In workshops with parallel sessions participants had the opportunity to choose among topics, for example PCC communication, and leadership and PCC. There was also designated time for HCPs from the same health care unit to discuss issues related to the operationalisation of PCC in their specific context. The learning seminars were run by staff at the Department for Development, invited researchers and clinicians with in-depth knowledge of PCC across Sweden, patient representatives, and HCPs from units in the region. The statement *support implementation of “more” PCC* was deliberately chosen and used by the staff at the Department for Development to increase HCPs buy-in to the innovation thereby acknowledging that PCC was to a certain extent adopted by some HCPs.

#### Support strategy and operationalisation of PCC at the unit level

After the learning seminars, each healthcare unit developed and executed its own strategy, designed to fit the particular context and working routines, in order to support implementation of more PCC. How PCC was operationalised at the units varied and included for example changes to the daily round in the ward to enable HCPs to talk with the patient rather than about the patient, changes to team compositions to improve care experiences for patients, and changes in the physical environment to enable undisturbed discussions with patients.

### Health care units

To gain a wide understanding of our research topic, we recruited participants from six separate healthcare units in the region [18, 19]. These units were a convenience sample based on participation in the learning seminars, a variety of healthcare settings, and the senior and frontline managers’ willingness to be part of the study and allow the research group access to collect data. The units represented: primary care, including rehabilitation and midwifery; psychiatric inpatient care for people with primarily depression and psychosis, where patients were admitted for both voluntary and compulsory care; geriatric in- and outpatient care at two hospitals; and nephrology outpatient care.

### Participants

Participants with various vocational roles were recruited from the six health care units. The inclusion criterion was HCPs working with patients at the units, moreover we purposely strived to include participants with different gender, ages, time at workplace and representing a variation of vocational roles found at each unit. Participants were recruited regardless of their participation in learning seminars or activities to support more PCC at the unit level. This choice was made to gain a broader perspective of views from HCPs representing implementation of PCC in a real-world setting, for example staff turnover, and ability/opportunity to attend learning seminars. Out of a total of 231 employees working with patients at these units, 97 participated in the study (see Table 1 for participant characteristics). Assistant nurses, registered nurses, and medical doctors were all represented in the interviews at all units. Allied HCPs were recruited in geriatric- and primary care units.

**Table 1:** HCPs characteristics (*n* = 97)

		*n*
Participants/total at units		97/231
Gender		
Female		80
Male		17
Employment at unit		
Years, mean (SD)	7.7 (10.9)	
Range, min/max	3 months – 42 years	
Profession		
Assistant nurse		30
Registered nurse		40
Medical doctor		10
Physiotherapist		9
Occupational therapist		7
Other		1

### Procedure

Interview data were collected in May and October 2018, during the implementation efforts at the different healthcare units (18 months after the third learning seminar at four healthcare units and 24 months later at two healthcare units). The manager of each unit approached HCPs and asked them to participate in the study. HCPs were given information about the purpose of the study, what participation entailed and its voluntary nature. Participation took place during HCPs’ workday, within their working hours. We combined focus groups [18], dyadic interviews [20], and individual interviews [21] in order to gain rich and varied data as well as for pragmatic reasons such as HCPs ability/opportunity to partake in focus groups at designated times. Focus groups (*n* = 15), dyadic interviews (*n* = 5), and individual interviews (*n* = 22) were conducted in secluded rooms designated by the manager of each unit, usually separate from the workplace. Focus groups [18] and dyadic interviews [20] were held with “pre-existing groups” at each unit, based on teams or vocational roles. Pre-existing groups were thought to ensure that participants could share, discuss, and compare their thoughts and experiences from one and the same context [18]. The first author, who acted as moderator/interviewer, had previous experience of interviewing and had undergone interview training during a doctoral course. She had formerly worked as a physiotherapist in the region and was well acquainted with the healthcare context but had not previously met any of the participants or visited the specific units included in the study. Either the second or third author was present during larger focus groups to act as notetakers and manage the audio recorder. The moderator asked participants to show respect for one another by not sharing anything that was said outside the group setting. Individual interviews were conducted to ensure that experiences and thoughts of a sensitive nature were expressed and included in the data [21]. Semi-structured interview guides were used with open-ended questions operating in the same fashion across all types of interviews. These targeted two major topics: participants’ perceptions of PCC in relation to their context and their perceptions of the strategies used to implement PCC. The findings for the first topic are reported in this article. The open-ended questions were not formulated to target predefined domains or constructs in the CFIR. The moderator/interviewer prompted participants to reflect on PCC in an introductory question asking about their first impressions, thoughts, and understanding of the concept, as well as if and how those impressions had changed across time. Key questions were then posed to explore how participants perceived PCC in relation to their practice, workplace, and routines, their perceptions of key factors that distinguish PCC from previous work, and if and how they perceived any changes in regard to patients, themselves, and/or the team if they worked more in line with PCC (Additional File 1 contains the interview guide for focus groups). The moderator/interviewer used member summaries throughout the focus groups and interviews to make sure she had correctly interpreted what participants said [21].

### Data collection

The number of focus groups, how many participants to include in each focus group, and the number of dyadic and individual interviews were guided by a pragmatic approach, i.e., participants’ working hours and number of employed healthcare professionals at each unit, as well as “no shows” due to high workload or illness. Focus groups consisted of three to eight participants [22]. Background variables on HCPs’ gender, vocational role, and duration of employment at each healthcare unit were collected before each interview. Interviews ranged from 20 to 77 min (focus groups mean 50 min, dyadic interviews mean 39 min, and individual interviews mean 40 min). All interviews were audio-recorded and transcribed verbatim. The moderator used field notes after each interview to reflect on the context, group dynamics, issues, and remarks made by participants in order to keep track of her own reflexivity [21]. Field notes were also used to make sure recruitment continued until no new information emerged. Quotes from the HCPs in the results section were translated from Swedish to English by the authors in collaboration with a native English speaker.

### Development of a coding sheet and data analysis

Qualitative content analysis was conducted using a deductive approach to apply the CFIR codes to the data, followed by an inductive analysis to describe participants’ perceptions of PCC in relation to their context [23]. The analysis was undertaken in NVivo using a stepwise approach by which the researchers moved forward and backward through the steps to validate, revise, and refine the findings [19]. In the first step, interview transcripts were read in full several times by the first author, who identified meaning units corresponding to HCPs’ perceptions of PCC. In step two, a few transcripts were initially reviewed by the research team to create a coding sheet with adapted definitions belonging to the CFIR domains and constructs designed to fit the study context and the complexity of the innovation (Additional File 2) [17]. The research group reviewed all the domains and constructs within the CFIR during this phase, as recommended by the founders of the CFIR [16]. Eight constructs were selected from the CFIR, six from the domain *Innovation characteristics* (innovation source, evidence strength and quality, relative advantage, adaptability, trialability, complexity). Cost was not discussed or described by HCPs in either the focus groups or the interviews, and design quality and packing are not included in this analysis because we interpreted that this construct would be strongly tied to strategies to support the implementation, which will be explored in a separate study. Two constructs were identified and selected from the domain *Inner setting* (compatibility, available resources). Moreover, the mixed deductive–inductive approach revealed that one construct, observability, emerged from the interview data, and this was added to the coding sheet. In several previous frameworks defining innovation attributes, *Observability* has been regarded as a construct of its own [13]. However, when the CFIR was first developed, *Observability* was integrated within the construct *Relative advantage* [16]. In our data, *Observability* emerged as a construct that had different and separate connotations from *Relative advantage* in relation to HCPs’ perceptions of PCC. The final coding sheet consisted of nine constructs with adapted definitions and coding criteria to facilitate the coding of the interview transcripts (Additional File 2). In step three, all the meaning units were analysed and coded deductively using the coding sheet. In step four, data belonging to each construct was analysed inductively and grouped into subcategories and generic categories. Throughout the inductive process, generic categories and subcategories were revised and refined. The deductive and inductive analysis was conducted by the first author in constant dialogue with the last author and in regular meetings with all members of the research group [24].

## Results

Five out of nine constructs on the coding sheet, *Relative advantage, Complexity, Compatibility, Observability,* and *Available resources* were discussed in depth by participants and resulted in a wide range of generic categories and subcategories. The other four constructs, *Innovation source, Evidence strength and quality, Adaptability,* and *Trialability* were represented in some of the interviews and discussed mostly at a surface level by participants. The CFIR constructs and key generic categories are summarised in Table 2 and reported below in the same order as they are described in the CFIR.

**Table 2:** Content analysis of focus groups, dyadic interviews, and individual interviews. Generic and subcategories generated using an unconstrained matrix with nine pre-defined main categories

Main categories	Generic categories
**Innovation Source**	Mixed perceptions about the origin of PCC
**Evidence strength and quality**	Improved health and systems outcomes in patients Lack of awareness of evidence underlining PCC
**Relative advantage**	In line with ethical values Improved work routines Sounds intuitively positive and stirs curiosity Identical to previous work Increased workload and deterioration of well-functioning routines
**Adaptability**	Adaptable to specific contexts
**Trialability**	Initial piloting to test applicability
**Complexity**	Abstract phenomenon that gives rise to conflicting views Leaves HCPs with ethical dilemmas and conflicting views Viewing and treating patients as persons is complex Requires a variation in skills and personal qualities in HCPs Requires integration within the team and between HCPs
**Compatibility**	Conflicting mixture of norms and values Contrasting perceptions of PCC routines and their fit with existing workflow Perceived similarities between PCC and other concepts increase compatibility
**Observability**	More satisfied and involved patients More meaningful, and improved work environment but also demanding Improved relationships and workflow within the team Mixed perceptions of work in team
**Available resources**	Requires overcapacity of resources to maintain PCC Physical environment can hamper or facilitate PCC Required resources dependent upon context and operationalisation of the concept

The extended coding tree with main categories (the CFIR constructs), generic categories, and subcategories is found in Additional File 3.

### Innovation source

The analysis demonstrates that HCPs voiced uncertainty and held mixed perceptions about the origins of PCC. These perceptions included beliefs that PCC, or its operationalisation, originated within their own unit, at an organisational level, or both. For example, one participant in a focus group noted that she was unsure if PCC, operationalised through changes to a team round, had been founded by two experienced colleagues:

*I don’t know if they [experienced colleagues] were the ones who came up with it?* (Focus group 7, inpatient care)

Some HCPs said they were unaware of the origins of PCC, and some said they were unaware that their healthcare unit had made the decision to work more in line with PCC.

### Evidence strength and quality

Among those participants who mentioned evidence of PCC, one comment that reappeared several times in the interviews was that working according to PCC would shorten patients’ hospital stay. One HCP gave a detailed description of improved lab results for patients who had participated in intervention studies in PCC. However, most comments from HCPs in this category were from participants who said they were unsure as to whether there was any strong evidence on PCC. This was exemplified by one HCP saying:

*I don’t think that, maybe that it gives any better results. Yeah, maybe in some places. With blood pressure and so on. Maybe it does.* (Interview 14, outpatient care)

### Relative advantage

The relative advantage of working in line with PCC, compared to how HCPs had worked before, was described from a range of different and sometimes conflicting perspectives. The results of the inductive analysis demonstrate that HCPs’ perspectives on PCC span a wide range of perceptions, from PCC as a self-evident and advantageous practice to a lack of awareness of PCC’s connotations or no perceived differences between PCC and previous work.

HCPs who were in favour of PCC discussed the perceived advantages from two main perspectives. First, PCC was perceived as being in line with their ethical beliefs and, second, the operationalisation of PCC into new routines was perceived to improve healthcare and their own working context. HCPs’ ethical beliefs were described as a striving for greater equity in care, and creating an environment that is inclusive for all patients in healthcare. Ethical perspectives were also expressed from HCPs’ own point of view, where they related PCC to their own experiences of being a patient, or as an imaginary patient describing how PCC resonates with how they themselves would like to be approached when they are or become patients.

Moreover, some HCPs who had recently graduated described being content that PCC was highlighted at their workplace, because it is in line with values that were propounded in their education.

*I thought, good God it’s like…weren’t we supposed to work like this? And why don’t we? In other words, it became a question for me... and that was scary. I’m trained to be a care expert and, in participation with the patient, be able to create care together.* (Interview 10, inpatient care)

The other perspective on relative advantage described by HCPs is the notion of how PCC had been operationalised into various clinical routines at the different units. Two units had operationalised PCC, amongst other routines, by shortening the time spent on their daily round to enable HCPs to have more time to spend together with patients. HCPs from these units perceived a major improvement in their workflow and said that it enabled closer contact between HCPs and patients.

A contrasting description in this category was that some HCPs were unable to see how PCC was any different from how they had worked before. Instead, PCC was just a new “fancy” word for something according to which they had always worked.

There were also reflections that PCC would lead to disadvantages in the workplace in relation to workload and routines. Some HCPs believed that changes to routines were made for the sake of changing something that already functioned well. An increased workload caused by the introduction of more routines was perceived as a waste of time for both the workplace and the HCPs.

### Adaptability

PCC in relation to the degree to which it can be adapted to fit local needs was narrated by HCPs from both the perspective of PCC as an innovation and when questioning whether their own contexts were thought to be compatible with the innovation. HCPs expressed the view that PCC could be seen as an innately flexible innovation because it should be based on individuals working and creating care together on a daily basis. Thus, some HCPs explained that they trusted in what patients perceived to be important for their care and tried to be flexible towards their needs, while others discussed how routines at their workplace could be changed to fit with their work context, exemplified by one HCP as:

*Well, for example, when we go to a multi-bed room with four patients, and you go and see someone and then they say: “No, I’d like to sleep a little longer.” Well then you can go to the next person. So, there’s a flexibility.* (Focus group 2, inpatient care)

### Trialability

New routines thought to be in line with PCC had been tried out on a smaller scale within different work teams or in a specific part of a ward. Decisions to test PCC on a smaller scale at a particular unit were made for several different reasons and from different perspectives. Some HCPs thought it was done to avoid scaring people off by introducing it first to those who seemed positive towards it, while others said it was to test it out to see how it worked before it was rolled out to all HCPs and patients. One participant recounted an experience of being in a team that was introduced to a routine at a later stage of the implementation:

*No, but it was care team one that started and, and we’re care team two. It flowed well for care team one with the [new round], and we still did our rounds, boring rounds, in the morning. So we thought, we also want to do that. So, we tried, and it went really well.* (Focus group 7, inpatient care)

Experiences of reversing course were also put forward by HCPs at three units that were trying to implement a bedside round as a means of including patients in all discussions about their health status. However, the bedside round was hindered by factors such as privacy issues, which arose because several patients shared the same room, and was therefore discontinued.

### Complexity

This category entails a range of conflicting descriptions from participants who depicted PCC as a highly complex innovation. The results of the analysis present PCC as an often vague and abstract phenomenon that carries different meanings for different individuals, exemplified by one HCP as:

*It’s hard because everyone creates an understanding of what person-centred care is. And then we’re supposed to go towards some sort of common idea, with like 40 different opinions on what it actually is. So there you have to start somewhere. We need to have some sort of common…we need to know how we’re going to work together first.* (Focus group 11, outpatient care)

Other HCPs talked about PCC in terms of how it had been operationalised, and their recollections were therefore based primarily on task issues. Some described finding it difficult to explain PCC in their own words and what the concept is about and entails in practice.

*Yes, it was hard to arrive at what we should do concretely. Like, it sounds like most [people] are interested in this way of thinking and think like this a lot, but what do you actually do to make it work?* (Focus group 4, inpatient care)

In contrast, other HCPs described feeling familiar with the connotations and meanings of PCC and its operationalisation to concrete routines, but concluded that they were aware that some HCPs in their own unit and others were not, thereby leading to a mismatch between different HCPs in terms of their understanding of PCC.

Complexity could not only be seen from the individual HCPs’ understanding of PCC but also in relation to each unique patient. The intricacies of working in partnership increased when HCPs met patients with communication difficulties, such as aphasia or speaking another language, when patients had different expectations of the care offered, and/or when patients did not want to work in partnership with HCPs. HCPs described feeling frustrated when some patients, described as being passive recipients of care, were uninterested in taking any active part in their care, planning, or decision-making. The analysis shows that working in partnership with patients was sometimes further complicated when relatives became involved. Relatives were primarily described from a dualistic perspective, whereby they could be seen as an important asset or as obstructing the partnership by demanding care or treatment that was not in line with the patient’s wishes. Another issue that emerged in the analysis was discussed in relation to HCPs working as a group or team. Descriptions were given of how PCC is all about changing HCPs’ mindset, such that everybody needs to learn how to think differently and in accord with one another. Sharing the same values was important but difficult for HCPs because contrasting norms and values existed in one and the same workplace. Some participants underscored that HCPs need to understand each other in the same way as they strive to understand each patient to enable more PCC. Thus, complexity was discussed in relation to each individual HCP, to each patient and sometimes their relatives, and finally to HCPs regarded as a group.

Another aspect of complexity was the ethical dilemmas to which PCC gave rise. Such dilemmas arise when there is a clash between a patient’s wishes and the evidence, or between a patient’s wishes and HCPs’ urge to do what they believe is best for patients. Some HCPs were confident in listening to the patient and making their wishes central, while others felt unable to let the patient make the decision. One ethical dilemma, discussed by participants from all units except the primary care unit, was related to patients not wanting to continue living. Among patients with conditions such as severe disabilities, depression, and old age, HCPs needed to navigate between the patient’s self-determination and the HCP’s own wish to do good, as well as workplace norms, rules, and regulations. An elaborate balancing act was described by HCPs when two conflicting wills had to be negotiated and resolved without creating too much conflict or making the patient feel steamrolled. Another ethical dilemma arose from experiences where decisions taken in partnership between an HCP and a patient do not always concur with the evidence base, which should be the basis for today’s healthcare. A clash between those loyalties was exemplified by one HCP as:

*It’s one of those quality indicators that gets published for everyone then, how things are in Sweden. And if you’re below average, then you’re ashamed, even though you really think you have individualised. The best way is to get good numbers, that is, to override the patients. There’s a conflict here. There’s a conflict between person-centred care and the national care values and guidelines.* (Interview 14, outpatient care)

Moreover, the analysis shows that PCC is also a demanding and complex innovation in relation to its operationalisation into skills and tasks. HCPs recognised that patients are individuals with different needs, wishes, and resources, and that HCPs need to have the skills to be sensitive to this. Participants reported and outlined a range of skills that HCPs need to embrace and learn, such as being a skilful listener, having a flexible attitude, being attuned to patients’ needs and wishes, and being able to consolidate difficult recollections from patients.

### Compatibility

Individual and group norms, values, and working routines from the workplace, in relation to PCC, emerged in this category. HCPs at several units stated that different teams at the same healthcare unit carried different norms amongst themselves. Thus, descriptions were made of groups of individuals being true and positive towards working in line with the values purported by PCC, while other groups of HCPs were described as being inflexible and trapped in a way of thinking that was not seen to be aligned with the underpinnings of PCC. This was exemplified by one participant in a focus group describing colleagues who were perceived as inflexible in relation to changing their work routines:

*Some patients want to sleep in the morning. No, everyone has to get up like at a certain time and like, no, you know, like, we wake XX and if there’s somebody who wants to have a little sleep in, then it’s really difficult, because it like upsets their entire rhythm for that day*. (Focus group 1, inpatient care)

Some HCPs said that PCC is natural and obvious in their context because they have always worked in line with these values, whilst others saw a genuine potential for improvement at their workplace regarding the delivering of more PCC. HCPs at all units associated PCC with other concepts and methods that they thought shared similar underpinnings, such as Motivational Interviewing and Comprehensive Geriatric Assessment, which were already part of their everyday practice. HCPs said that they were unsure of the differences between these various concepts and PCC. Moreover, HCPs’ perceptions that they were already working in a manner similar to PCC was regarded by some HCPs as a positive aspect that strengthened their perceptions of the compatibility of PCC with their existing workplace routines.

### Observability

Recollections were offered of changes in patients, within HCPs themselves, and within the team when work was or was not in line with PCC. Positive changes were observed in patients as they became more independent, were calmer, trusted HCPs with more and more sensitive information, and told HCPs that they felt listened to when PCC was in place. When PCC was not in place, HCPs described how patients lost their self-esteem and became passive. This was recounted by one participant:

*They become a shadow. Somebody goes in and takes over and decides everything for the patient. And then you lose your own self, I think. Then you don’t have the patient along with you, they give up. And if they don’t have any self-confidence and so on, then it becomes, then they become only a shadow, you could say.* (Interview 2, inpatient care)

Moreover, working in line with PCC was observed as being more fun and creating better flow in the workplace. The analysis reveals feelings of satisfaction, meaningfulness, and personal connectedness with patients, along with decreased feelings of insufficiency when HCPs described their experiences of listening to patients’ narratives and talking about their lives and wishes.

In contrast, some HCPs described how working in line with PCC is more exhausting and can create feelings of inadequacy when stress at the workplace or stringent routines hinder PCC practices. Teamwork in conjunction with PCC was distinguished as something that glued teams together, leading to improved relationships between team members. However, this was contradicted by some HCPs, who recounted that they had not observed any changes within the team.

### Available resources

A common discussion amongst HCPs in the interviews were statements regarding resources in relation to PCC. HCPs were adamant that a prerequisite for working in line with PCC is to have appropriate resources available. Such resources were discussed in terms of personnel, physical space, and time. This was exemplified by one HCP saying:

*If you work with people, not machines, it can…it takes time with people. That’s how it is. And when you sit down and interview and find out what it’s about, then you need to have more time, and not have this stress.* (Focus group 13, outpatient care)

PCC was described as having different connotations in relation to time. Some HCPs were adamant that it takes more time to work in line with PCC, while others recounted that it takes less time. Some stated that their workplace was well suited for working in line with PCC and that no extra resources were needed to change to more PCC. HCPs who worked in a context where they had fixed and sufficient time to meet with patients concurred that they did not perceive a lack of time to work in line with PCC. However, PCC in relation to available resources, especially in an inpatient context, was expressed as difficult to maintain on a constant basis due to a slimmed-down organisation that falters when there is a high workload or a lack of personnel. Participants who worked at two healthcare units where PCC had been operationalised into a different round, targeting especially the time component, described a complete change in their work context and stated that they perceived having more time to spend listening and talking to patients. HCPs working in an inpatient context described how some chores, like washing a patient, are more time efficient in the short run, in comparison to assisting the patient to do it on their own, thereby highlighting a conflict between perceived time consumption and PCC. However, HCPs also noted that patients who were supported to become more independent in their everyday activities would need less assistance and this would save time for HCPs in the long run. Some HCPs also felt that their work to support patients to become more independent would not lead to any evident time savings at their unit, but instead these would manifest when patients finally moved back to their homes or into other caring facilities.

## Discussion

We have explored 97 HCPs’ perceptions of PCC in relation to their own context through the lens of the CFIR. These HCPs worked in a wide range of vocations and in a variety of contexts, which may increase the transferability of the results to other settings. At the extremes, we encountered some HCPs who were well acquainted with the concept, had participated in learning seminars, and had taken extra-curricular courses in PCC. At the other extreme, some HCPs stated that they had not received any information about PCC and did not even know that they were supposed to work in line with this concept.

The results of the analysis show that PCC was perceived as a highly complex innovation with different connotations, specifically in relation to two perspectives that were sometimes distinct and separate and sometimes interrelated. First, PCC seen through the lens of its ethical underpinnings where HCPs discussed PCC as a way of being with patients regardless of different work tasks. In this case, the ethics of seeing the person and getting to know more about their thoughts and values was underscored. Second, PCC regarded from the perspective of its operationalisation into concrete working routines where HCPs talked exclusively about PCC in relation to concrete work tasks that they perceived to be in line with PCC. Activities such as discussing goals from the patient’s perspective, letting patients shower more often, and writing a health plan are examples of these concrete work tasks. HCPs at different units sometimes gave similar descriptions of tasks, whilst other tasks pertained solely to a single unit. Viewing PCC from these perspectives is in line with earlier research on PCC, where the founders of different schools of PCC underscore the importance of integrating its ethical underpinnings and its operationalisation into working routines [7, 15, 25, 26]. Our findings also reveal that HCPs’ perceptions of PCC as an innovation need to be considered from this dualistic perspective in order to gain a broad understanding of the determinants of practising PCC. It is noteworthy that these two perspectives on PCC are represented both separately and in conjunction with one another throughout the deductive constructs in the CFIR and in the categories that emerged from the inductive analysis.

Another prominent finding that emerged from the analysis in relation to the complexity of PCC is the notion voiced by HCPs representing all units that they do not know what PCC is or entails, or what they are supposed to do to work in line with PCC. Descriptions were given of PCC being perceived as an abstract concept that was difficult for many HCPs to grasp. An innovation is defined by Rogers as “an idea, practice, or object that is perceived as new by an individual or other unit of adoption” p.12 [27], and similar definitions are found in other implementation frameworks that include constructs related to innovation [1]. Thus, a prerequisite for HCPs who are supposed to adopt PCC would naturally be the understanding of what PCC is, what it entails in practice, and the extent to which they perceive the concept as a new approach at their workplace. The results of the analysis, depicting PCC as an abstract concept without clear operationalisations, becomes quite problematic when considered in conjunction with implementation research pointing to the importance of specifying and describing innovations in order to make them usable for the adopters [28, 29]. It seems an impossible challenge for HCPs to adopt something that some say they are unaware of or do not understand.

The analysis shows that the CFIR construct *Compatibility* is related to a range of factors, such as work tasks, vocational roles, and HCPs’ previous experience related to concepts they perceive as similar. In this category, it becomes clear that PCC is indeed a profoundly complex innovation, with some HCPs working in the same workplace and sharing the same vocational roles describing how they have different perspectives on PCC and its compatibility with the workplace. Thus, HCPs working within the same unit described quite different perceptions of PCC. This is partially in line with other studies of HCPs’ perspectives on PCC describing diverse and sometimes conflicting perceptions [15]. In one of these studies, three factors were highlighted that specifically influenced variations in perceptions towards a PCC intervention. Unclear objectives within the intervention and unclear roles of mandate between HCPs constituted two of the factors. The third factor was tied to vocational roles and work tasks and showed a lack of inter-vocational understanding of PCC [15]. The findings of our study do not support the notion that perceptions of PCC are tied to vocational roles but suggest rather that they are based on individual HCPs being unique in their attitude towards PCC. This is in line with results from a recent study focusing on patients’ perceptions of PCC carried out at the same units as those described in this study [30]. Patients’ perceptions of PCC in relation to different HCPs were dependent on the individual characteristics ascribed to each unique HCP, and not based on vocational roles [30].

The results of this analysis identify a range of resources that are perceived as being important to enable PCC. Having enough staff capacity to accommodate situations of increased stress, for example, was essential for maintaining PCC at all times. Similar results have been found in other research on PCC, where job strain and a supportive psychosocial climate were the most important factors related to variations in PCC [31]. Resources are highlighted as imperative in teachings about PCC and in the newly launched European Standard, which aims to support the implementation of a minimum level of PCC across Europe [3, 7, 25]. Moreover, having the correct resources in place before implementation efforts commence is also seen as a prerequisite in a range of implementation frameworks, models, and theories [1]. However, the results of the current study show that the resources needed to work in line with PCC may differ across contexts, vocational roles, and work tasks, and as such need to be examined in conjunction with each of these factors.

Providing care according to current evidence on healthcare interventions and clinical expertise, while simultaneously remaining in line with each patient’s values and circumstances, is often advocated as best practice and promoted as evidence-based practice [32]. Current research evidence, clinical expertise, and PCC may be regarded as interrelated without any inherent conflicts. In contrast, the results of this study, in the CFIR construct *Complexity*, show that current research/clinical evidence and PCC are sometimes viewed as opposing concepts that lead to ethical dilemmas, as described in detail by many HCPs in this study. They concurred that they want to do what is “right” but were hesitant as to what that may be in some situations and with some patients. They were torn between adhering to patients’ wishes and aspirations, and following guidelines or other care documents based on research evidence. PCC is based on the notion of integrating patients’ lived experiences, expertise, and wishes for care with current research evidence and clinical expertise [7, 8, 25]. Our findings show that HCPs sometimes struggle to navigate between these perhaps inherently conflicting demands. Similar findings have been reported in previous studies showing the challenges that arise for clinicians supporting patient self-care [33], HCPs trying to incorporate biomedical perspectives into the lived experiences of patients [34], and HCPs feeling restricted by traditional care pathways and standardised prescribing when they are trying to work according to PCC [35]. We believe that these ethical dilemmas warrant further research and illustrate the importance of increased understanding and reflection between HCPs when patient needs and wishes, different circumstances, resources, care, evidence, rules, and regulations represent conflicting values. The notion of encouraging regular meetings among HCPs for reflection and evaluation of work carried out is often emphasised in studies discussing the teachings of PCC [7, 25]. Such evaluative reflection meetings could perhaps act to bridge the gap between the conflicting values that HCPs face when they are trying to work according to PCC in a natural and everchanging context.

### Methodological considerations

Content analysis employing a mixed deductive–inductive approach using constructs from the CFIR to describe HCPs’ perceptions of PCC in relation to their context was chosen for three overarching and long-term reasons [16, 19, 36, 37]: to facilitate the comparison of results from multiple and repeated data collections across time; to enable comparisons of results from other research studies based on the CFIR domains and constructs; and to increase in-depth knowledge about PCC from the HCPs’ point of view in order to gain insights that can serve to tailor the implementation of PCC in the future [17].

We decided to consider all the constructs available in the CFIR in order to address the study’s aim and to justify our selection of some of these constructs in our coding sheet [17]. The construct *Knowledge and beliefs about the innovation* might at a first glance seem to fit with our aim, which is to explore HCPs’ perceptions of PCC in relation to their context. However, we argue that, to gain a thorough understanding of a complex innovation and guide future efforts to implement PCC, there is a clear advantage to separating HCPs’ perceptions into more detailed constructs that can be acted upon, with matching strategies, at a later stage. Moreover, we believe that *Relative advantage* may well be regarded as an overarching construct that is the tipping point when HCPs add up all the pros and cons depicted in the other constructs in the CFIR, compare these with current practice, and then decide whether or not to adopt the innovation.

The founders of the CFIR acknowledge that the boundaries between domains and constructs are dynamic and sometimes difficult to discern from one another [16]. We discussed all of the CFIR constructs in the research group and decided to use the code *Compatibility* for statements regarding PCC and its fit with existing workflows, tasks, and values. Statements based on HCPs’ perceptions of working with PCC were in turn coded to the construct *Complexity*. We adapted the CFIR by adding *Observability* to our deductive coding sheet. Participants described clearly how the results of working with PCC were visible to themselves and others thereby distinguishing observability from relative advantage. Future studies will need to be conducted to validate this adaptation to the CFIR. Moreover, we did not use the construct *Patient needs and resources* that is found in the CFIR domain *Outer setting*. The reason for this was that the innovation of PCC, per se, incorporates the patient as a prerequisite, a central and crucial part of the actual innovation. Our choice can be contrasted with that of Safaeinili et al., who described an evaluation of a patient-centred care transformation within a learning health system [38]. They chose instead to adapt the CFIR by creating a new, sixth domain entailing the construct *Patient needs and resources* to incorporate a better fit with their data and study aim [38].

### Limitations and strengths

In this study we chose to mix focus groups, with dyadic and individual interviews to gain rich and varied data from the participants and to increase participants’ possibility of partaking in the study at various times and dates according to their work schedules. Mixing interview formats is sometimes questioned as different formats target different foci [39]. We used focus groups and dyadic interviews to spur discussions and reflections between participants and individual interviews to increase data richness ensuring that experiences and thoughts that may be sensitive to share in a focus group e.g., not sharing the same norms or values as colleagues, were not lost. The interview guides, with their open-ended questions, were not developed to capture the specific constructs in the CFIR. The results may therefore depict a data collection bias whereby some constructs in the CFIR may appear less prominent than others in relation to PCC. As PCC has been acknowledged to be a highly complex innovation, we did not want to lock ourselves into preconceived ideas of what factors to consider in relation to it. Not using predefined questions based on the CFIR constructs could be argued to have given participants more freedom to share their perceptions of PCC, thereby minimising confirmation bias of the CFIR constructs. The study used a cross-sectional design and we can therefore only report HCPs perceptions of PCC at one point in time.

## Conclusion

The findings of this study support previous studies describing PCC as a highly complex innovation with a wide variation in how PCC is viewed by HCPs. Robust knowledge of factors that are important for the implementation of an innovation is required in implementation processes in clinical practice and in implementation research. The results should not be regarded as a blueprint of HCPs’ perceptions of PCC, but rather as a valuable contribution discussing factors that may be important to consider in a wide range of contexts within the healthcare sector. HCPs’ perceptions of PCC are shaped by a range of factors, such as their individual understandings of the concept and the operationalisation of PCC in their local context. By gaining an in-depth understanding of HCPs’ perceptions of PCC, stakeholders in charge of implementation processes can target perceived barriers linked to the innovation, and thus improve the opportunities of successful implementation. Moreover, HCPs’ perceptions of PCC, described through the lens of the CFIR, can act as a valuable asset for future studies seeking to compare and aggregate findings from other settings where the implementation of PCC is explored.

## Supplementary Information

**Additional file 1.** Interview guide focus groups.

**Additional file 2.** Code sheet with CFIR constructs, general definitions and adapted definitions for the IMPROVE project. Observability added as a new construct.

**Additional file 3.** Content analysis of focus groups, dyadic interviews, and individual interviews. Generic and subcategories generated using an unconstrained matrix with nine pre-defined main categories (CFIR constructs).

## Data Availability

The datasets used during this study are available from the corresponding author on reasonable request.

## Abbreviations

HCP: Healthcare professional
PCC: Person-centred care
GPCC: The Gothenburg University Centre for Person-Centred Care
CFIR: The Consolidated Framework for Implementation Research
