# Supplementary material for: [The innovation characteristics of person-centred care as perceived by healthcare professionals: an interview study employing a deductive-inductive content analysis guided by the consolidated framework for implementation research
Source: BMC Health Serv Res. 2021 Sep 3;21:904. doi: 10.1186/s12913-021-06942-y (PMC8414852; doi:10.1186/s12913-021-06942-y)
Supplement: Supplementary file 1 — Additional file 1. Interview guide focus groups. [file 12913_2021_6942_MOESM1_ESM.pdf]

**Additional file 1. Interview guide focus groups.**

**Interview guide: Focus groups with HCPs (English*)**

*This is a translation from Swedish, provided for publication purposes and might not represent a validated translation.

Thank you for participating today in a group discussion about the introduction of more person-centred care at your workplace. You have been asked to participate in this group discussion because we are interested to learn more about your thoughts on person-centred care and your experiences of work carried out at your workplace to introduce more person-centred care.

**Presentation of myself and the project:**

Introduction of myself, my supervisor, and the project. Previous work in clinical care, research, and my current position as a doctoral student at Dalarna University.

Project background: The discussions we will have today are part of a larger project aimed at increasing knowledge about the processes that take place in a healthcare context when new knowledge is implemented within an organisation. We want to understand what happens in a natural setting when work is carried out by people in the organisation and not orchestrated by researchers. What kind of work is carried out to introduce the knowledge, how do HCPs perceive these actions, and what do the patients think? We are following six units in the region, and you are employed at one of them. We are collecting data in many different forms and today we are conducting a group interview.

**Introduction, about the group discussion:**

The group discussions will be recorded and then transcribed to a text file. All information from this focus group will be kept in a locked location at Dalarna University. Only people engaged in the research group will have access to this information. Your colleagues and managers at your workplace will not have access to the transcripts from your group discussion and when we report the results of this project it will not be possible to identify any individuals. To ensure that everybody in the group feels free to speak their own mind, I ask you not to repeat what is being said in this group discussion. However, you are not legally bound to silence, and I ask you to be aware of this. Participation in the interview is voluntary and you may end your participation at any time, without giving any reason for doing so. I am interested in all your thoughts, both large and small, on the questions. There are no right or wrong answers, and this is not a test of your knowledge about PCC. If there are parts of your answers that I don't quite understand, I will ask you to explain what you mean so that I am sure to interpret your input and discussions correctly.

**Questions:**

1. Can we start off by going round and all of you telling me your first name and how long you have been working in this workplace?
2. Can you tell me about your initial thoughts and perceptions of person-centred care when you first heard of the concept?
   Follow up: Have your thoughts changed over time as you have worked to introduce more PCC at your unit?
3. Can you perceive a difference between PCC and your "old" way of working?
4. What are the characteristics of PCC, are there any special signs or actions?
5. What are your expectations of PCC? What will be different? In what way? For the patient? Yourselves? The team?
6. What are your discussions about PCC like at your workplace?
7. Round up with an overall conclusion of the discussions about PCC and then ask: is there anything that we have missed in relation to your thoughts and perceptions on PCC and that you think it is important to add? Please tell me.
8. We will now move on and talk about activities that you have been using, aimed at introducing more PCC into your workplace. (These questions are not reported in this publication.)

**Prompts:**

Can you tell me more?

Can you give me an example?

Do you have anything more to add?

Do you mean that…… [moderator summarises the content of the discussion]?

Does that mean that…… [moderator summarises the content of the discussion]?

If I try to summarise/interpret what you just discussed…… [moderator summarises the content of the discussion]? Did I get it right?
