# Supplementary material for: [The innovation characteristics of person-centred care as perceived by healthcare professionals: an interview study employing a deductive-inductive content analysis guided by the consolidated framework for implementation research
Source: BMC Health Serv Res. 2021 Sep 3;21:904. doi: 10.1186/s12913-021-06942-y (PMC8414852; doi:10.1186/s12913-021-06942-y)
Supplement: Supplementary file 2 — Additional file 2. Code sheet with CFIR constructs, general definitions and adapted definitions for the IMPROVE project. Observability added as a new construct. [file 12913_2021_6942_MOESM2_ESM.pdf]

Additional file 2. Code sheet with CFIR constructs, general definitions and adapted definitions for the IMPROVE project. Observability added as a new construct.

Construct	General definition	Adapted definition
**Innovation Source**	Perception of key stakeholders about whether the intervention is externally or internally developed	HCPs perceptions of about whether PCC has been developed externally or internally.<br><br>**Inclusion criteria:** Include statements related to the origins of PCC as well as actions and routines developed to operationalise PCC in the health care setting.<br>**Exclusion criteria:** Exclude statements related to HCPs perceptions regarding leadership engagement.
**Evidence strength and quality**	Stakeholders' perceptions of the quality and validity of evidence supporting the belief that the intervention will have desired outcomes	HCPs perceptions of the quality and validity of evidence supporting the belief that PCC will have desired outcomes.<br><br>**Inclusion criteria:** Include statements related to knowledge about scientific research AND recollections from colleagues at other health care units.<br>**Exclusion criteria:** Exclude statements related to perceived observed benefits from working more in line with PCC and code to Observability.
**Relative advantage**	Stakeholders' perception of the advantage of implementing the intervention versus an alternative solution.	HCPs perception of the advantage of implementing PCC versus an alternative solution i. e. working as before.<br><br>**Inclusion criteria:** Include statements related to perceptions of the advantage OR disadvantage OR no change of working more in line with PCC compared to how work has been conducted before. Include statements that are related to patient needs and resources as this is directly tied to the innovation.<br>**Exclusion criteria:** Exclude statements related to HCPs perceived pros and cons of PCC that can be coded to any of

		the other constructs in the framework. i. e complexity issues, compatibility to own workplace.
**Adaptability**	The degree to which an intervention can be adapted, tailored, refined, or reinvented to meet local needs.	HCPs perception of the degree to which PCC can be adapted/tailored to their local needs.<br><br>**Inclusion criteria:** Include statements about perceived adaptability related to the origins of PCC as well as actions and routines developed to operationalise PCC in the health care setting.<br>**Exclusion criteria:** Exclude statements made in relation to different activities and tasks that were tried out and code to Trialability.
**Trialability**	The ability to test the intervention on a small scale in the organization, and to be able to reverse course (undo implementation) if warranted.	HCPs perceptions of the ability to test working more in line with PCC i.e. the operationalisation through actions and routines in the health care setting and perceived possibility to be able to reverse course (undo implementation) if warranted.<br><br>**Inclusion criteria:** Include statements related to different activities and tasks that were tried out/piloted at the workplace.<br>**Exclusion criteria:** Exclude statements related to adaptations of activities and tasks to fit with HCPs local needs and code to Adaptability.
**Complexity**	Perceived difficulty of implementation, reflected by duration, scope, radicalness, disruptiveness, centrality, and intricacy and number of steps required to implement.	HCPs perception of the degree of complexity involved to work in line with PCC.<br><br>**Inclusion criteria:** Include statements related to complexities of working in line with PCC. See reference Grol et al. "the degree to which a new working method is considered to be difficult to understand, multifaceted and/or awkward to use".

		**Exclusion criteria:** Exclude statements related to complexity of implementation.
**Compatibility**	The degree of tangible fit between meaning and values attached to the intervention by involved individuals, how those align with individuals own norms, values and perceived risks and needs, and how the intervention fits with existing workflows and systems.	HCPs perceptions in relation to the degree of tangible fit between meaning and values attached to PCC by involved individuals, how those align with individuals own norms, values and perceived risks and needs, and how the intervention fits with existing workflows and systems.<br><br>**Inclusion criteria:** Include statements related to existing routines, and perceived values based on individual expressions and the values and norms perceived at the workplace.<br>**Exclusion criteria:** Exclude statements related to PCC as being difficult to understand or use and code to complexity.
**Observability**	See reference Rogers " the degree to which the results of an innovation are visible to others".	HCPs perceptions of the apparent or visible benefits of working more in line with PCC.<br><br>**Inclusion criteria:** Include statements based on perceptions regarding HCPs observations of what they perceive at the workplace when more or less PCC is delivered.<br>**Exclusion criteria:** Exclude statements based on hearsay from other health care professionals or scientific research and code to evidence strength and quality
**Available resources**	The level of resources dedicated for implementation and on-going operations, including money, training, education physical space, and time.	HCPs perceptions of resources needed to work in line with PCC.<br><br>**Inclusion criteria:** Include statements based on HCPs perceptions on resources needed at the workplace to be able to work in line with PCC.<br>**Exclusion criteria:** Exclude statements related to PCC in relation to perceived complexities and code to complexity.
