# Supplementary material for: [The innovation characteristics of person-centred care as perceived by healthcare professionals: an interview study employing a deductive-inductive content analysis guided by the consolidated framework for implementation research
Source: BMC Health Serv Res. 2021 Sep 3;21:904. doi: 10.1186/s12913-021-06942-y (PMC8414852; doi:10.1186/s12913-021-06942-y)
Supplement: Supplementary file 3 — Additional file 3. Content analysis of focus groups, dyadic interviews, and individual interviews. Generic and subcategories generated using an unconstrained matrix with nine pre-defined main categories (CFIR constructs). [file 12913_2021_6942_MOESM3_ESM.pdf]

Additional File 3. Content analysis of focus groups, dyadic interviews, and individual interviews. Generic and subcategories generated using an unconstrained matrix with nine pre-defined main categories (CFIR constructs).

Main categories	Generic categories	Subcategories
**Innovation Source**	Mixed perceptions about the origin of PCC	Developed within the unit<br>Developed by the organisation<br>Developed by the organisation and within the unit
**Evidence strength and quality**	Improved health and systems outcomes in patients	Shortened hospital stays<br>Research has shown improved health outcomes
	Lack of awareness of evidence underlining PCC	Unaware of evidence base
**Relative advantage**	In line with ethical values	Equal care for patients<br>In line with values taught in education<br>Improved quality of care<br>How one would like to be approached if a patient oneself
	Improved work routines	Improved routines with clear instructions<br>More time with patients due to changed routines<br>Boost work towards same goals<br>Improved contact with patients<br>Allows flexibility to each patient's needs
	Sounds intuitively positive and stirs curiosity	Sounds positive without knowing what it entails<br>Excited and curious to know more
	Identical to previous work	No difference, always worked this way<br>A new and fancy word for something that we already do<br>Tied more strongly to some vocational roles and settings
	Increased workload and deterioration of well-functioning routines	Changing routines that are perceived to already work<br>More workload for an already tired workforce<br>Different views on relative advantage based on vocational roles
**Adaptability**	Adaptable to specific contexts	Flexible methods and solutions for integrating PCC<br>More easily adapted to some contexts than others
**Trialability**	Initial piloting to test applicability	Initiated in parts of the unit<br>Starting on a small scale so as not to scare HCPs<br>Tried routines that were abandoned
**Complexity**	Abstract phenomenon that gives rise to conflicting views	A vague construct that is difficult to grasp<br>Conflicting understandings amongst HCPs

		Difficult to explain and unclear what it entails in practice<br>A vast concept that entails everything in healthcare<br>Easier to accommodate and endorse than HCPs think
	Leaves HCPs with ethical dilemmas and conflicting views	Patient's wishes lead to ethical dilemmas<br>Routines can both aid and hamper PCC<br>Prioritising when resources are scarce<br>Conflicting views between relatives' and patient's wishes
	Viewing and treating patients as persons is complex	Difficulties in communication complicates partnership<br>Patient expectations and changes in power relationships makes partnership complex<br>PCC works with all patients<br>Relatives' involvement ambiguous
	Requires a variation in skills and personal qualities in HCPs	Being able to accommodate difficult narratives<br>Acting as a role model<br>Being compassionate, attentive and a skilful listener<br>Having a flexible attitude<br>Being skilled in communication with patients<br>Being skilled in communication within and across organisations<br>Capturing the person's life world<br>Being skilled in including the person's perspective in documentation
	Requires integration within the team and between HCPs	Developing a deeper understanding of each other<br>Sharing the same values within the team<br>Having regular discussions and reflections within the team<br>Creating a feeling of togetherness with the patient<br>Working towards the same goals<br>Bringing new team members into the approach
**Compatibility**	Conflicting mixture of norms and values	In line with own norms and values<br>Working similarly before<br>Norms described as being divergent within and across units
	Contrasting perceptions of PCC routines and their fit with existing workflow	Distinctions between own and other HCPs' performance within and across units<br>Choosing between set routines and staying flexible to patient needs<br>Great potential to increase PCC routines<br>PCC disturbs routines that are firmly in place

	Perceived similarities between PCC and other concepts increase compatibility	Motivational interviewing<br>Terma<br>Comprehensive Geriatric Assessment<br>Patient-focused care
**Observability**	More satisfied and involved patients	Patients report that they feel listened to and are satisfied with care<br>Patients are more involved in their care<br>Less anxious patients<br>Patients reach goals and are discharged sooner<br>More trustful relations<br>Patients lose their self-esteem and become hospitalised when work is not in line with PCC
	More meaningful, and improved work environment but also demanding	Work more satisfying and meaningful<br>Better flow, safer and less stressful<br>More trust in patient's own capacity<br>Exhausting to listen to patients' narratives<br>Ethical stress when work situation does not enable PCC
	Improved relationships and workflow within the team	Stronger and tighter team<br>Less stress and more fun within the group<br>Increased humility between team members
	Mixed perceptions of work in team	No perceived changes in the team<br>Various levels of changes in different teams
**Available resources**	Requires overcapacity of resources to maintain PCC	Difficult to maintain PCC when short of staff<br>Difficult to maintain PCC when patients need more time-consuming care than normal at the unit<br>Feelings of stress make it difficult to accommodate principles of PCC
	Physical environment can hamper or facilitate PCC	Secrecy issues with several patients in multi-bed room<br>More time efficient when patients are in multi-bed room<br>Physical distance between team members complicates PCC teamwork
	Required resources dependent upon context and operationalisation of the concept	Saves time in the long run<br>More time consuming<br>Less time consuming<br>More, well-educated staff
